# Virulent and Avirulent Strains of *Toxoplasma gondii* Which Differ in Their Glycosylphosphatidylinositol Content Induce Similar Biological Functions in Macrophages

**DOI:** 10.1371/journal.pone.0085386

**Published:** 2014-01-28

**Authors:** Sebastian Niehus, Terry K. Smith, Nahid Azzouz, Marco A. Campos, Jean-François Dubremetz, Ricardo T. Gazzinelli, Ralph T. Schwarz, Françoise Debierre-Grockiego

**Affiliations:** 1 Institute for Virology, Laboratory of Parasitology, Philipps-University, Marburg, Germany; 2 UPR 9022 CNRS, Institute of Molecular and Cellular Biology, Strasbourg, France; 3 Biomedical Sciences Research Complex, University of St Andrews, St Andrews, United Kingdom; 4 Research Center René Rachou, Oswaldo Cruz Foundation, Laboratory of Immunopathology, Belo Horizonte, Brazil; 5 UMR 5235 CNRS, University of Montpellier 2, Montpellier, France; 6 Department of Biochemistry and Immunology, Institute of Biological Sciences, Federal University of Minas Gerais, Belo Horizonte, Brazil; 7 Division of Infectious Diseases and Immunology, Department of Medicine, University of Massachusetts Medical School, Worcester, Massachusetts, United States of America; 8 UMR 8576 CNRS, Unit of Structural and Functional Glycobiology, University of, Lille, France; 9 UMR 1282 Infectiology and Public Health, University of Tours, Tours, France and INRA, Nouzilly, France; University of Wisconsin Medical School, United States of America

## Abstract

Glycosylphosphatidylinositols (GPIs) from several protozoan parasites are thought to elicit a detrimental stimulation of the host innate immune system aside their main function to anchor surface proteins. Here we analyzed the GPI biosynthesis of an avirulent *Toxoplasma gondii* type 2 strain (PTG) by metabolic radioactive labeling. We determined the biological function of individual GPI species in the PTG strain in comparison with previously characterized GPI-anchors of a virulent strain (RH). The GPI intermediates of both strains were structurally similar, however the abundance of two of six GPI intermediates was significantly reduced in the PTG strain. The side-by-side comparison of GPI-anchor content revealed that the PTG strain had only ∼34% of the protein-free GPIs as well as ∼70% of the GPI-anchored proteins with significantly lower rates of protein N-glycosylation compared to the RH strain. All mature GPIs from both strains induced comparable secretion levels of TNF-α and IL-12p40, and initiated TLR4/MyD88-dependent NF-κBp65 activation in macrophages. Taken together, these results demonstrate that PTG and RH strains differ in their GPI biosynthesis and possess significantly different GPI-anchor content, while individual GPI species of both strains induce similar biological functions in macrophages.

## Introduction

The obligate intracellular protozoan *Toxoplasma gondii* ranks among the most common parasites of animals and humans, with an estimated worldwide incidence of 30% of chronically infected individuals [1]. The canonical lineages, designated types 1, 2, 3 (formerly I, II, and III) and the haplogroup 12, comprise the genus *Toxoplasma* in Europe and North America [2], [3], [4]. These haplogroups differ genetically by 1% or less but diverge in a number of phenotypes such as growth, migration potential, and most notably acute virulence in laboratory mice [5], [6]. Type 1 strains are highly virulent and uniformly lethal with LD_100_ of <10 parasites, whereas type 2 strains are avirulent in most, but not all, inbred mouse lines with a median lethal dose (LD_50_) of ∼10^3^ parasites. Type 3 strains are typically avirulent with LD_50_≥10^5^ parasites [7], [8].

*Toxoplasma* polymorphic effector molecules subvert key host defense mechanisms and account for much of the strain-specific difference in virulence in mice [9], [10]. These are introduced during invasion into the host cytosol from secretory compartments, namely rhoptries and dense granules, and traffic to the host nucleus and to the nascent parasitophorous vacuole membrane (PVM). The high virulence of type 1 strains is thus largely due to the rhoptry protein (ROP)18 kinase, the closely related ROP5 pseudokinase, and the ROP16 kinase [6], [11], [12], [13], [14]. The former kinases target host immunity by disrupting the function of immunity-related GTPase (IRG)-mediated parasite clearance in macrophages, while this process is not affected by type 2 and 3 strains, where IRGs cause disruption of the PVM and subsequent parasite destruction [15], [16], [17]. The latter alters host gene transcription through sustained activation of signal transducer and activator of transcription (STAT)3/STAT6 and promotes alternative macrophage activation (M2), thereby limiting protective Th1 responses, which in turn favor parasite growth, causing possibly a secondary response that leads to cytokine-mediated pathology [18], [19], [20], [21]. This detrimental overstimulation of host immunity is likely triggered by parasite components, such as profilin, cyclophilin-18, and glycosylphosphatidylinositol (GPI) anchors [22], [23], [24].

GPI anchors are common glycolipids in all eukaryotes that mainly function to covalently anchor surface proteins to membranes, while synthesized at much higher rates in parasitic protozoa than in other eukaryotes [25]. Over the last two decades many biological and immunological properties have been found associated with GPI anchors and GPI-related molecules of protozoan parasites [26], [27], [28]. Both protein-free GPIs and/or several GPI-anchored proteins, including SAG1-related sequence proteins (SRS) and SAG-unrelated surface antigens (SUSA) have been reported to decorate the surface of all *T. gondii* life cycle stages, however the molecular function and biological importance of most surface proteins remain poorly defined [29], [30], [31], [32], [33]. The majority of known SRS and SUSA proteins have dual functions, either as adhesins mediating initial host cell attachment, and/or immune decoys eliciting or modulating inflammatory responses during acute infection [29], [30], [31], [33]. The GPI biosynthesis has been shown to be essential for *T. gondii* viability but it remains unknown whether the observed lethality is due to the lack of protein-free GPIs, GPI-anchored proteins or both [34]. The protein-free GPI intermediates from the virulent *T. gondii* RH strain have been structurally analyzed and demonstrated to induce a TLR2/TLR4/MyD88-mediated TNF-α production in macrophages that is dependent on the expression of galectin-3 [23], [35], [36], [37]. In contrast, little was known about the structure and the biological functions of GPIs from avirulent *T. gondii* strains.

In this report, we characterized the GPI content and assessed the biological function of individual GPI species of an avirulent type 2 strain (PTG). We demonstrated that this strain possesses significantly less GPI content, lower levels of GPI-anchored proteins, and lower rates of GPI-anchored protein N-glycosylation than the virulent RH strain, while protein-free GPIs of both strains activate identical TLR signaling pathways and induce comparable levels of inflammatory cytokines in macrophages.

## Materials and Methods

### Analysis, extraction, and purification of individual GPIs

GPIs of the strains RH and PTG of *T. gondii* were metabolically labeled by incubating parasites grown in *Mycoplasma*-free Vero (American Type Culture Collection, ATCC) cells, in 20 ml glucose-free DMEM containing 20 mM sodium pyruvate and supplemented with 0.5 mCi D-[6-^3^H]-glucosamine hydrochloride (Hartmann Analytic GmbH, Braunschweig) for 6 h at 37°C in 5% CO_2_ atmosphere. Remaining intracellular parasites were released from host cells with the help of glass beads in a Mixer Mill homogenizer (Retsch, Haan) and subsequently purified from host cell debris by glass wool filtration [38]. Labeled and unlabeled GPIs were purified from a determined number of parasites using the method previously described to obtain both protein-free and GPI-anchored proteins [39]. Briefly, glycolipids were extracted with chloroform-methanol-water 10∶10∶3 (v/v), partitioned between water and water-saturated butan-1-ol, and precipitated gently under a nitrogen stream. Finally, GPIs recovered from the butan-1-ol phase were separated by thin-layer chromatography (TLC) on Merck Si 60 HPTLC plates using chloroform-methanol-hexane-water-acetic acid 10∶10∶3∶2∶1 (v/v) as solvent and D-[6-^3^H]-glucosamine labeled GPIs used as tracers. Chromatograms were scanned for radioactivity using a Berthold LB 2842 linear analyzer and areas corresponding to individual GPIs [35] were scraped off, re-extracted with chloroform-methanol-water 10∶10∶3 (v/v), and recovered in the butan-1-ol phase after water-saturated butan-1-ol/water partition to obtain individual purified GPIs. The absence of endotoxin in purified GPIs was validated with the *Limulus* Amebocyte Lysate kit QCL-100 (Bio-Whittaker, Walkersville, MD). GPIs were stored at −20°C in butan-1-ol until use.

### Structural analysis of GPI core glycans

Extracted GPIs were dephosphorylated, deaminated, and reduced as described elsewhere [40]. Briefly, GPIs were dephosphorylated with 48% aqueous hydrofluoric acid for 60 h at 0°C and the reaction was stopped by blowing off hydrofluoric acid under a nitrogen stream. Molecules were suspended in 400 µl of freshly prepared 0.1 M sodium acetate (pH 3.5) containing 0.25 M NaNO_2_ and incubated at room temperature (RT) for 4 h. The reaction was then terminated by addition of 300 µl of 0.4 M boric acid and 130 µl of 1 M NaOH. The material was reduced overnight at 4°C using 2 M NaBH_4_ in 0.3 M NaOH and the reaction was stopped with 5% acetic acid in methanol that was removed by two evaporations with 50 µl toluene. The resulting material was desalted on a Chelex 100 (Na^+^) - AG50X12 (H^+^) - AG3X4 (OH^−^) - QAE Sephadex A25 (OH^−^) ion-exchange column and filtered through a 0.2 µm filter. D-[6-^3^H]-glucosamine-labeled neutral glycans were digested by 2 units of Jack bean α-mannosidase or N-acetylhexosaminidase in 100 µl of 50 mM sodium acetate (pH 4.5) containing 0.2 mM ZnCl_2_ and 0.02% sodium azide for 24 h at 37°C [41]. Analysis was performed by HPAEC using a Bio-LC Dionex module (Dionex Corporation, Sunnyvale, CA) before and after digestion. Samples were mixed with 0.6 mg of partially hydrolyzed dextran [42] prior to injection into a Carbopac® PA1 anion-exchange column equilibrated with 100 mM NaOH. Elution was accomplished with 100% buffer A (100 mM NaOH) for 6 min, followed by a linear increase of buffer B (100 mM NaOH, 0.25 M NaOAc) from 0 to 30% in 30 min at a flow rate of 1 ml/min. Positions of glucose monosaccharide and oligomer standards were obtained using pulsed amperometric detection, while eluted radiolabeled glycans were detected by scintillation counting.

### GPI quantification and carbohydrate composition analysis

The method is based upon quantification of the GlcN residues of the GPIs being converted to AHM as described elsewhere [43] with slight modifications. An internal standard of *scyllo*-inositol was added to each triplicate sample containing either protein-free GPIs or GPI-anchored proteins. Controls with various GlcN and *myo*-inositol concentrations were prepared and processed in parallel. Samples were dephosphorylated, deaminated, reduced, and transferred to glass capillaries in 100 µl methanol-water 1∶1 (v/v) and dried in a vacuum concentrator. Methanolysis was conducted by adding 50 µl of dry methanol containing 0.5 M HCl, flame sealing the capillaries under vacuum and incubating them at 95°C for 4 h. The glass capillaries were opened, pyridine (10 µl) was added to neutralize the HCl and re-N-acetylation was performed with acetic anhydride (10 µl) for 30 min at RT. Samples were dried twice from dry methanol (20 µl) in a vacuum concentrator. Derivatization was performed with 10 µl of TMS (trimethylchlorosilane-hexamethyldisilazane-dry pyridine, 1∶3∶10, v/v) for 20 min prior to analysis of 2 µl of the products by GC-MS on a MS detector-5973 (GC-6890N, Agilent Technologies, Santa Clara, CA) with a HP-5 column (30 m×25 mm) at 80°C for 2 min followed by a gradient up to 140°C at 30°C/min and a second gradient up to 265°C at 5°C/min and held at 265°C for a further 10 min. Ion monitoring of m/z 273 was selected to detect AHM and m/z 318 to detect both *scyllo*- and *myo*-inositol. The peak areas of the corresponding standards were used to calculate the molar relative response, allowing quantification of both AHM and *myo*-inositol in the samples. The same method was used to determine monosaccharide content. Analyses were conducted in triplicate on 0.5 nM of *myo*-inositol containing glyconjugate/glycoprotein, with 1.0 nM *scyllo*-inositol as an internal standard. A mixture of sugar standards (each at 0.5 nM) was run in parallel to determine the molar relative response factor.

### Structural analysis of PIs

Extracted GPIs were subjected to deamination, followed by organic extraction prior to analysis by ES-MS on a Quattro Ultima triple quadrapole instrument. MassLynx was used to record and process the data [44].

### Immunofluorescence microscopy

HFF (ATCC) cells were grown to confluence on glass coverslips and infected either with *T. gondii* RH or PTG strains. Cells were fixed (48 h post infection, h p.i.) with 4% paraformaldehyde and subsequently permeabilized with 0.02% Triton-X 100, 10% bovine serum albumin (BSA) for 30 min, washed three times with PBS, and incubated for 1 h in 0.02% Triton-X 100, 10% BSA with mAb T54 E10 [45] that recognizes the EtN-PO_4_ and GlcGalNAc epitopes of protein-free GPIs [35]. Cells were washed again for three times with PBS and incubated for 1 h with secondary FITC-conjugated anti-mouse immunoglobulin antibody (DakoCytomation, Glostrup) in 0.02% Triton-X 100, 10% BSA. Finally, cells were washed three times with PBS and mounted in Fluoprep (BioMérieux, Marcy-l'Etoile).

### Cytokine measurements

RAW 264.7 (ATCC) and J774 (ATCC) mouse macrophages were used to measure TNF-α or IL-12p40 levels, respectively. Macrophages were incubated with protein-free GPIs purified by TLC from the RH or PTG strain. For that purpose, the GPI material needed was dried under a stream of nitrogen to volatilize the butan-1-ol. The GPIs were suspended by sonication in PANSERIN 401 serum-free medium. Measurements of TNF-α and IL-12p40 in cell culture supernatants were determined using a specific sandwich ELISA (BD Pharmingen), following the manufacturer's directions. GPIa, a chemically synthesized GPI molecule of *T. gondii* [46] kindly provided by Prof. Richard R. Schmidt (Department of Chemistry, University of Konstanz, Germany), was tested on J774 cells.

### NF-κB p65 transcription factor assay

RAW 264.7 macrophages were stimulated for 15, 30, and 60 min with or without protein-free GPIs purified from 4×10^8^ parasites of the PTG strain or with medium alone. Macrophage proteins were extracted and activation of NF-κB was measured using the TransAM™ NF-κBp65 transcription factor assay kit according to the manufacturer's instructions (Active Motif, Rixensart).

### FACS analysis

The CHO reporter cell lines (CHO/CD14, expressing endogenous functional TLR4; 7.19/CD14/TLR2, expressing stably transfected human TLR2; and the 7.19 clone, expressing neither TLR2 nor functional TLR4) contain a *cd25* gene reporter under the control of the human E-selectin promoter, which contains an NF-κB-binding site [47], [48]. The CHO reporter cell lines were generated as described elsewhere [48]. Cells were plated at 1×10^5^ per well in 24-well tissue-culture dishes. After 18 h stimulation with medium alone or with protein-free GPIs purified from 1×10^9^ parasites of the PTG strain, cells were stained with PE-labeled anti-CD25 (mouse mAb to human CD25, PE conjugate; Caltag Laboratories, Burlingame, CA), examined by FACS analysis and evaluated with CellQuest software (BD Biosciences, San Jose, CA).

### Statistical analysis

An unpaired Student t-test was used for statistical evaluation and P≤0.05 was considered statistically significant.

## Results

### PTG and RH strains synthesize different sets of protein-free GPIs

**Figure 1:**
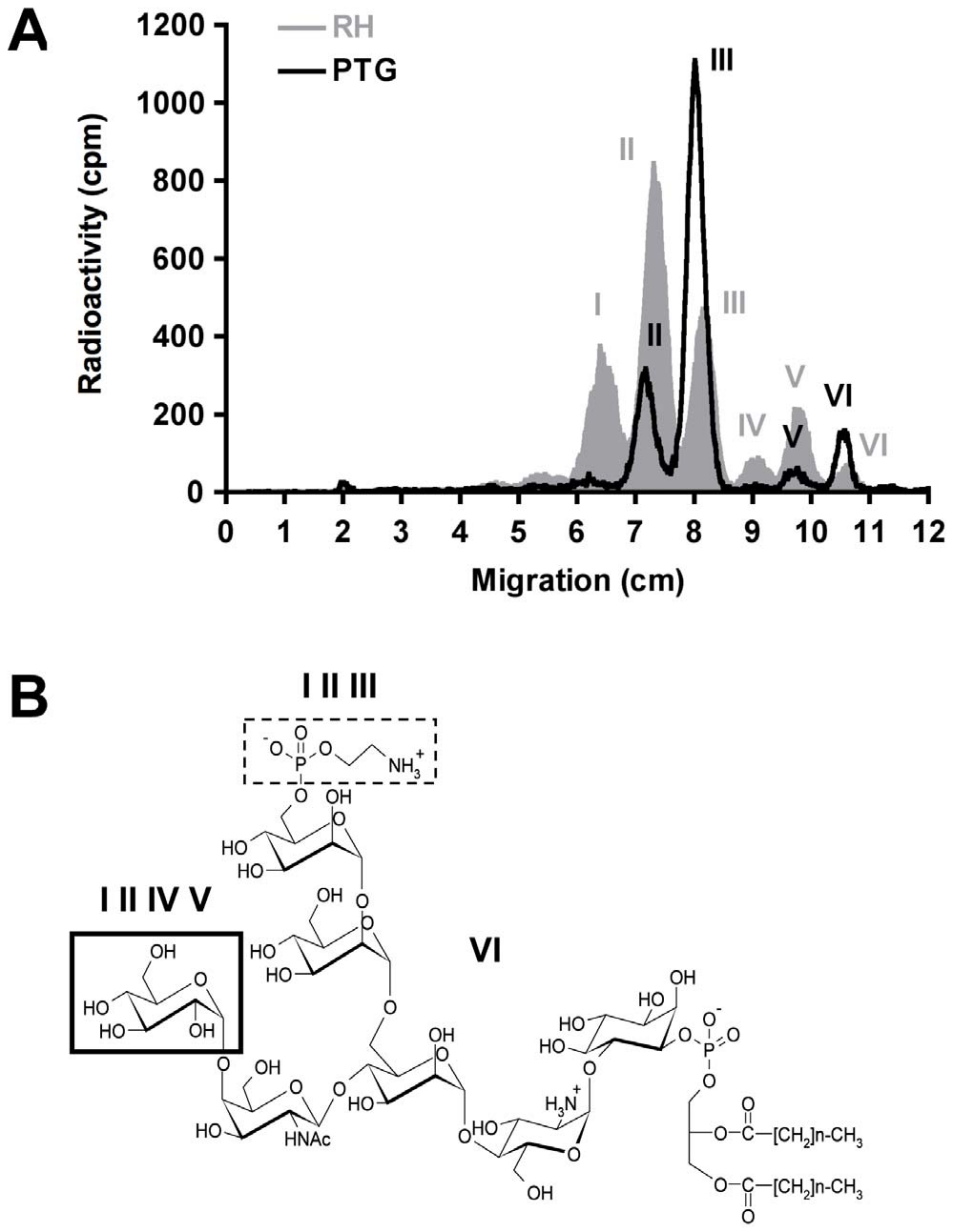
RH and PTG strains have different GPI profiles. (**A**) Metabolically labeled ([^3^H]-glucosamine) parasite glycolipids were extracted and separated by TLC as described in Materials and Methods. TLC chromatograms were scanned for radioactivity using a Berthold LB 2842 linear analyzer. Six different peaks representing individual GPIs are detected in the RH strain [35], whereas only four major peaks are present in the PTG strain. (**B**) Structures of *T. gondii* RH GPIs. GPI VI (Man-Man-[GalNAc]Man-GlcN-PI) is neither substituted with an EtN-PO_4_ nor with the Glc linked to the GalNAc residue. GPIs I, II and III contain an EtN-PO_4_ residue (dashed frame line). GPIs I, II, IV and V are substituted with a Glc that is linked to the GalNAc side-branch in α1–4 linkage (thickened frame line). GPIs I, II and IV, V, respectively, have identical carbohydrate moieties and differ in their fatty acid composition.

We first sought to examine whether the virulent RH strain and the avirulent PTG strain synthesize identical GPI structures. To this end, we metabolically labeled these two strains with D-[6-^3^H]-glucosamine. Figure 1A shows the separation of [^3^H]-labeled protein-free GPIs of both strains by thin layer chromatography (TLC). The RH strain synthesized six different GPI structures with previously described structures [35]. In contrast, the PTG strain synthesized only four major GPIs that seem to correspond to GPIs II, III, V and VI of the RH strain. The Glc-GalNAc-substituted GPIs I and IV were strongly reduced to near background levels in the PTG strain (Figure 1A). The peaks of both strains migrated with nearly identical Rf (rate of flow) values, suggesting only minor differences in their structures (Figure 1A).

### GPIs of PTG and RH strains have similar core glycan structures

**Figure 2:**
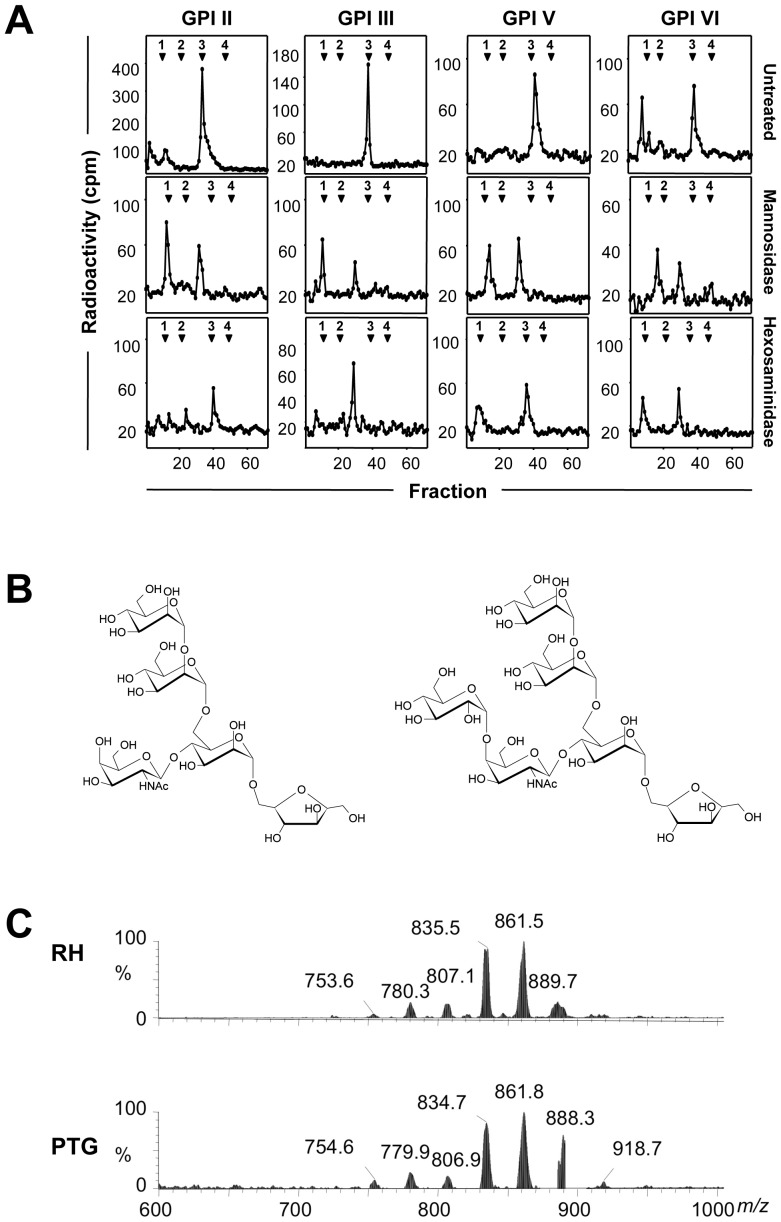
GPI core glycans and PIs of RH and PTG strains have similar structures. (**A**) HPAEC analysis of the core glycans generated from *T. gondii* PTG strain protein-free GPIs II, III, V and VI. TLC-purified D-[^3^H]-glucosamine labeled GPIs were dephosphorylated, deaminated and reduced. The resulting neutral glycans were analysed before (untreated) and after Jack bean α-mannosidase or hexosaminidase treatments. The elution positions of the co-injected glucose oligomer standards are indicated at the top of each profile (bold digits) and given as DU. (**B**) Predicted Man-Man-(Glc-GalNAc)Man-anhydromannitol core glycan structure derived from protein-free GPIs II and V (right panel), and predicted Man-Man-(GalNAc)Man-anhydromannitol core glycan structure derived from protein-free GPIs III and VI (left panel) of the PTG strain. (**C**) ES-MS spectra of the PI moieties released by deamination from purified protein-free GPIs from RH (upper panel) and PTG (lower panel) strains.

To investigate whether the protein-free GPIs synthesized by the PTG strain are structurally similar to those of the RH strain, we first analyzed their core glycan structures. The GPIs II, III, V and VI of the PTG strain were individually subjected to dephosphorylation, deamination and reduction to generate Man_3_-anhydromannitol (AHM) core glycans. They were then analyzed by high-performance anion-exchange chromatography (HPAEC) together with an internal dextran hydrolysate standard (dextran unit, DU). All four core glycans co-eluted with the 3 DU standard (Figure 2A, upper panel), similar to those of the RH strain [35]. To detect the presence of α-D-mannosyl residues, core glycans were individually digested with α-mannosidase. Core glycans individually treated with α-mannosidase eluted at 2.4 DU instead of 0.9 DU, the elution position of AHM (Figure 2A, middle panel). This incomplete digestion suggests the presence of a non-mannose substituent linked to the glycan cores, which may represent N-acetylgalactosamine (GalNAc) bound to GPI core glycan structures of the RH strain [35]. The presence of GalNAc residues on PTG strain core glycans was assessed by N-acetylhexosaminidase digestion. The core glycans derived from GPIs II and V were resistant to digestion with N-acetylhexosaminidase (elution at 3 DU, Figure 2A, lower panel), whereas core glycans derived from GPIs III and VI were sensitive to this treatment (elution of Man_3_-AHM at 2.5 DU and of GalNAc at 1 DU, Figure 2A, lower panel). This suggests the presence of an extra residue that is linked to the GalNAc, thus hindering its enzymatic removal by N-acetylhexosaminidase, which is similar to GPIs II and V of the RH strain that have an additional Glc residue [35]. We propose that the GPIs of the PTG and RH strains have similar GPI core glycan structures, namely Man-Man-(Glc-GalNAc)Man-anhydromannitol for GPIs II and V, and Man-Man-(GalNAc)Man-anhydromannitol for GPIs III and VI (Figure 2B).

### GPIs of RH and PTG strains have similar PI structures

We next analyzed the structures of the phosphatidylinositols (PI) moieties of the protein-free GPIs from both RH and PTG strains by negative ion electrospray mass spectrometry (ES-MS). The ES-MS analysis revealed that the PI moieties from both strains were very similar, with the vast majority of the heterogeneous PI species containing a *sn*-2 C18∶1 acyl chain (Figure 2C, and Table S1). The two most abundant PI species found in both RH and PTG strain GPIs were C34∶1 (C16∶0, C18∶1; 835 m/z) and C36∶2 (C18∶1, C18∶1; 861 m/z). The only significant difference was the increased abundance of the PI species at 889 m/z, C38∶2 (C20∶1, C18∶1) in GPIs of the PTG strain (Figure 2C). This difference in heterogeneity was also observed in PI moieties obtained from the protein-anchoring GPIs of both strains (data not shown).

### Glc-GalNAc-substituted GPIs of RH and PTG strains are exclusively present on extracellular parasites

**Figure 3:**
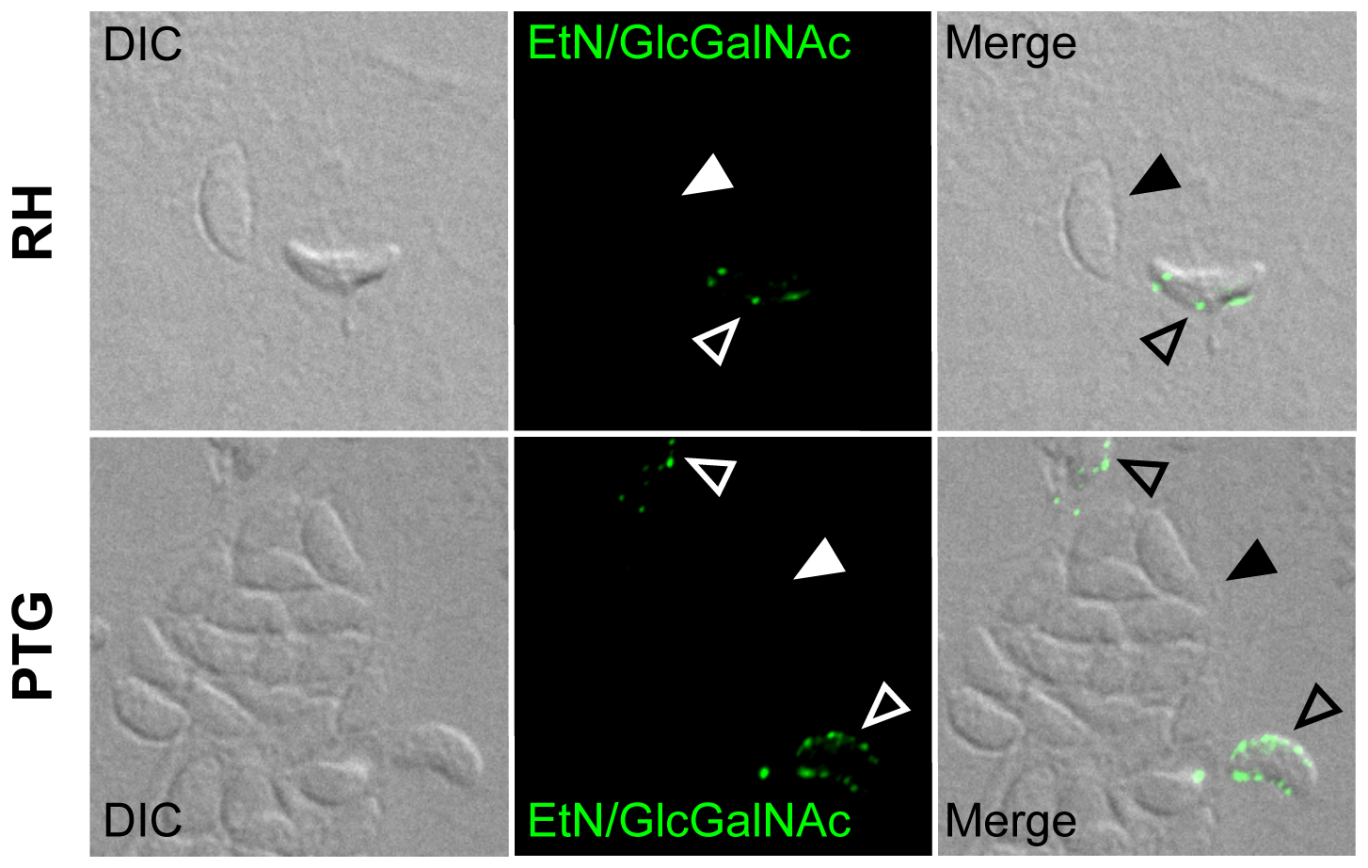
Protein-free Glc-GalNAc-substituted GPIs are clustered on extracellular parasites. Staining after permeabilization of HFF cells infected (72 h p.i.) with RH (upper panel) and PTG (lower panel) strains was performed using mAb T54 E10, recognizing both the EtN-PO_4_ and the Glc-GalNAc side-branch epitopes of protein-free GPIs. Filled arrowheads point to intracellular parasites residing inside parasitophorous vacuoles. Unfilled arrowheads point to extracellular parasites. DIC, differential interference contrast.

We have previously shown that the Glc-GalNAc-substituted GPIs remain protein-free and clustered in detergent resistant membrane domains (DRMs) that are enriched in cholesterol and sphingolipids, whereas the GalNAc-substituted GPIs are used to anchor surface proteins in RH strain parasites [32]. To investigate the distribution pattern of protein-free Glc-GalNAc-substituted GPIs in the PTG strain, we performed parasite labeling with mAb specific for the EtN-PO_4_ and the Glc-GalNAc side-branch of GPIs. Figure 3 illustrates that protein-free Glc-GalNAc-substituted GPIs are present on parasites of the PTG strain with similar distribution pattern and to the level found on the surface of RH strain parasites. Interestingly, we could not detect any labeling specific for protein-free Glc-GalNAc-substituted GPIs on intracellular parasites, whatever the strain type. This could be due to an insufficient permeabilization procedure before labeling. To exclude this possibility, the labeling procedure was repeated with both intracellular parasites mechanically released from their host cells and extracellular parasites harvested from cell culture supernatants. This approach confirmed that the Glc-GalNAc-containing GPIs are exclusively found on extracellular parasites (Figure S1). The labeling of the main surface protein SAG1 confirmed the accessibility and integrity of parasite surface molecules (Figure S1).

### RH strain has more protein-free GPI and protein-anchored GPI content than the PTG strain

To evaluate quantitative difference between GPIs of both strains, we analyzed both protein-free GPIs and protein-anchored GPIs of RH and PTG strains by gas chromatography-mass spectrometry (GC-MS). The method is based upon quantification of the GlcN residues of the GPIs being converted to AHM [43]. The close correlation between the AHM and the *myo*-inositol content of the GPIs indicated a high quality of the extraction and purification methods employed (Table 1). This analysis revealed that the PTG strain had about 70% of the total amount of GPI-anchored proteins of the RH strain, while the total protein-free GPI content was reduced to 34%, most probably due to the missing GPI I and IV. This quantitative reduction of GPI-anchor content in the PTG strain may lead to a reduction of SRS and/or SUSA proteins. Non-quantitative peptide-mass fingerprinting analysis of the GPI-anchored proteins identified identical SRS proteins, namely SAG1, SAG2, and SRS3 in both strains (Table S2) despite different SDS/PAGE profiles of GPI-anchored protein fractions of both strains (Figure S2). Together, the quantitative comparison of AHM revealed that the RH strain has significantly higher protein-free GPI, as well as protein-anchored GPI content than the PTG strain.

**Table 1:** Comparison of AHM and inositol quantifications of protein-free GPIs and GPI-anchored proteins of RH and PTG strains.

Sample	AHM quantification^a^	Inositol quantification^a^
RH protein-free GPIs	4.25±0.21×10^6^	4.54±0.16×10^6^
PTG protein-free GPIs	1.46±0.13×10^6^ (34%)	1.84±0.22×10^6^ (41%)
RH GPI-anchored proteins	1.12±0.11×10^6^	1.42±0.26×10^6^
PTG GPI-anchored proteins	0.78±0.04×10^6^ (70%)	1.02±0.07×10^6^ (72%)

a Copies per cell. In parentheses are percentages of PTG values compared to RH values. Values are means ± SD of three replicates.

### GPI-anchored proteins of the RH strain have a higher degree of N-glycosylation than GPI-anchored proteins of the PTG strain

To determine whether the glycosylation rates of protein-free GPIs and GPI-anchored proteins correlate with the GPI content, we compared protein-free GPIs and GPI-anchored proteins of both strains for altered glycosylation levels by GC-MS. The carbohydrate composition analysis of protein-free GPIs of the RH and PTG strains revealed a molar ratio of Ino∶Man∶GlcNAc∶GalNAc of 1∶3∶2∶1 and 1∶3∶1∶1, respectively (Table 2). Significant higher molar ratios of glucose were detected in protein-free GPIs of the RH strain (Table 2). This is consistent with the TLC-analysis of metabolically labeled PTG strain GPIs that demonstrated that two of four Glc-GalNAc-substituted GPIs were dramatically reduced in this strain (Figure 1A). We also found unexpected presence of galactose, fucose, and xylose in protein-free GPIs with higher molar ratios for the RH strain. The latter may be evidence of co-purification of an as yet unidentified glycoconjugate or a minor contamination by a GPI-anchored protein, which is N- and/or O-glycosylated. The analysis of RH and PTG GPI-anchored proteins revealed an average of N- and possibly O-glycosylation with higher molar ratios of mannose and GlcNAc in RH GPI-anchored proteins (Table 2). These results suggest that the RH strain possesses a significantly higher degree of N- and possibly O-glycosylation in GPI-anchored proteins than the PTG strain.

**Table 2:** Carbohydrate composition analysis of protein-free GPIs and GPI-anchored proteins of RH and PTG strains.

Sample	Ino	Man	GlcNAc	GalNAc	Gal	Glc^a^	Fuc	Xyl
RH protein-free GPIs	1.0±0.1	3.9±1.1	2.5±0.7	1.2±0.3	2.6±1.4	5.6±2.2	2.5±0.3*	1.2±0.1**
PTG protein-free GPIs	1.0±0.1	3.2±0.4	1.6±0.3	0.7±0.2	0.4±0.1	2.3±0.7	0.8±0.2	0.6±0.1
RH GPI-anchored proteins	1.0±0.1	18.9±3.2*	7.3±0.6*	1.4±0.3	6.3±1.2	14.1±3.9	7.6±0.1	8.0±1.0
PTG GPI-anchored proteins	1.0±0.1	9.3±2.6	4.5±1.1	1.2±0.1	4.1±1.9	15.1±4.1	5.5±0.5	7.2±0.8

a Glucose is a common contaminant. Values are means of molar ratios ± SD of three replicates.

Values are means of molar ratios ± SD of three replicates.

** P<0.005,

* P<0.05 RH protein-free GPI and GPI-anchored protein samples compared to PTG protein-free GPI and GPI-anchored protein samples, respectively.

### Protein-free GPIs of RH and PTG strains stimulate comparable levels of TNF-α and IL-12 secretion

**Figure 4:**
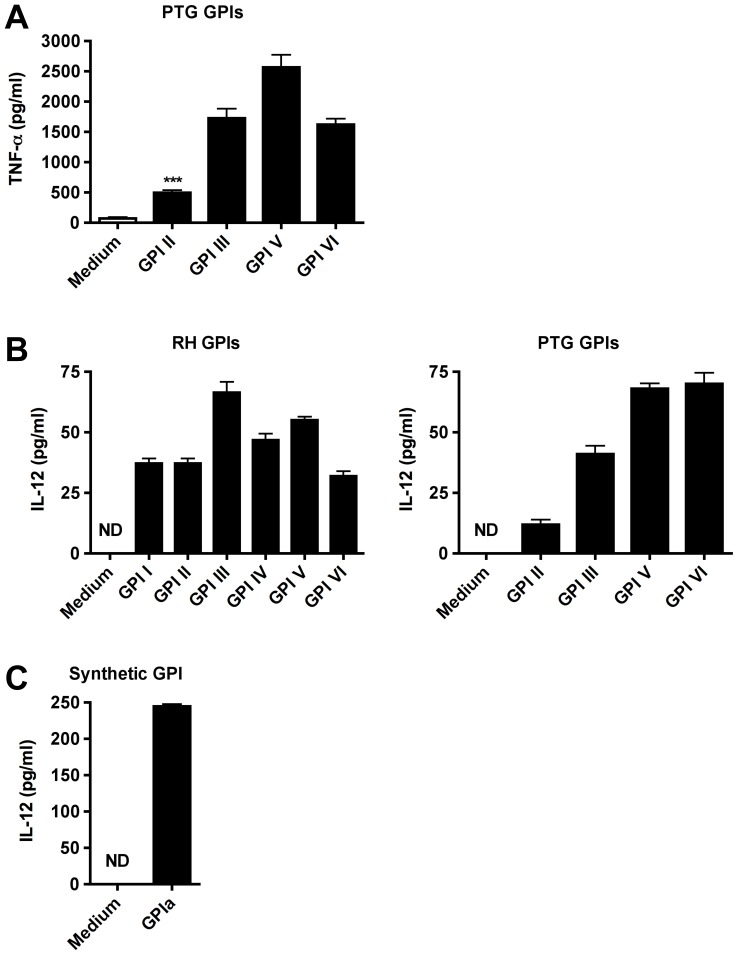
GPIs of RH and PTG strains induce TNF-α and IL-12p40 secretion by macrophages. (**A**) Macrophages were incubated for 24 h with medium alone, or with individual GPIs of the PTG strain extracted from 1×10^8^ parasites and assayed for TNF-α cytokine production. (**B**) Macrophages were incubated for 24 h with medium alone, or with individual GPIs of the RH (left panel) and the PTG strain (right panel) extracted from 2×10^8^ parasites, respectively, and assayed for IL-12p40 cytokine production. (**C**) Macrophages were incubated for 24 h with medium alone, or with GPIa (3 mM), a chemically synthesized structure of RH strain GPI III core glycan and assayed for IL-12p40 cytokine production. ^***^P<0.0005 PTG GPI II compared to medium control. ND, not determined.

The six protein-free GPIs of the RH strain have been shown to stimulate macrophages to secrete TNF-α [23]. To measure the biological activity of the four protein-free GPIs of the PTG strain, we individually added them to macrophage cultures. All four PTG GPIs induced significantly the secretion of TNF-α by macrophages (Figure 4A) to a similar extent than those of the RH strain [23]. An early production of IL-12 is essential for the host resistance against *T. gondii* in mice and only few parasite-derived molecules have been identified to induce IL-12 production [49], [50]. We therefore tested whether the protein-free GPIs of both strains could induce the secretion of IL-12 by macrophages. Figure 4B shows that all GPIs purified from RH (left panel) and PTG (right panel) strain parasites induced significantly the secretion of IL-12 by macrophages. Importantly, glycolipids extracted from uninfected host cells as well as silica alone did not stimulate any cytokine secretion, suggesting that this activity is specific for *T. gondii* GPIs [23]. To further validate whether the IL-12 production is specifically induced by GPIs and not due to a contaminating molecule, we incubated macrophage cultures with GPIa [(EtN-PO_4_)-Manα1-2Manα1-6 (Glcα1-4GalNAc β1-4)Manα1-4GlcNα-inositol-PO_4_], a chemically synthesized structure of RH strain GPI III core glycan. GPIa induced the production of IL-12, thereby demonstrating that the production of IL-12 in response to GPIs is specific and not due to a contaminating molecule (Figure 4C). In sum, these data suggest that RH and PTG GPIs have similar inflammatory capacities due to similar structures.

### Protein-free GPIs of the PTG strain induce TLR4-dependent NF-κB activation

**Figure 5:**
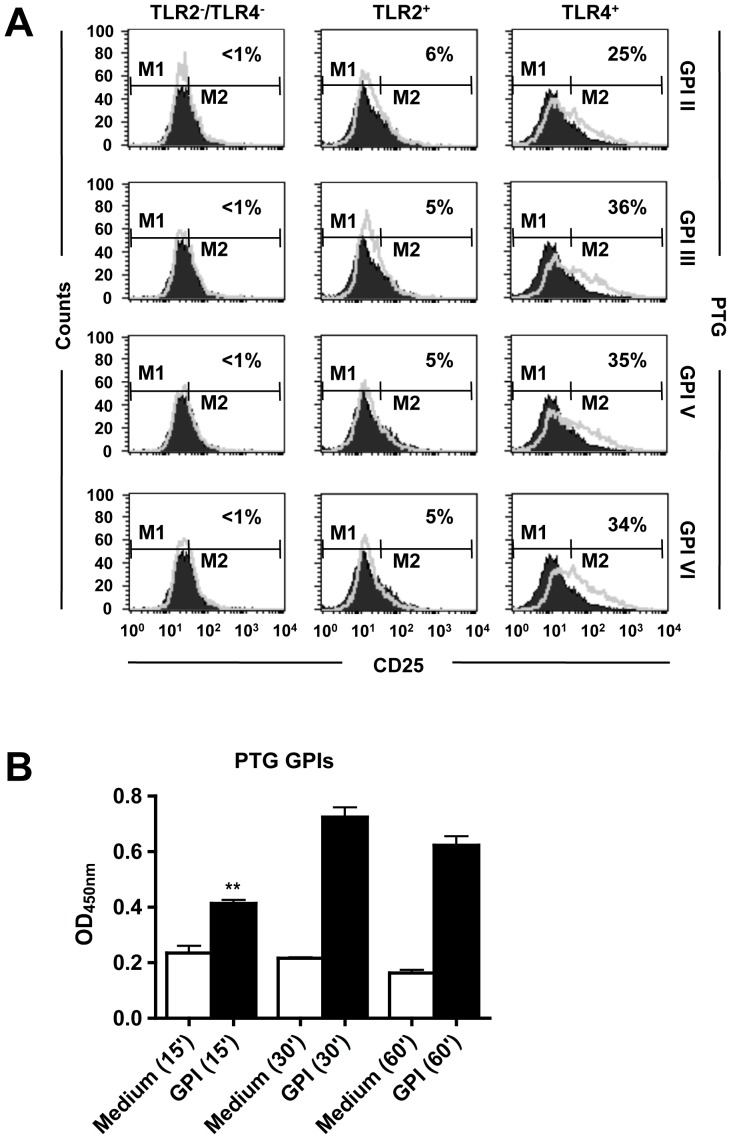
GPIs of the PTG strain activate TLR4/NF-κB signaling. (**A**) CHO cells expressing TLR2 (TLR2^+^), TLR4 (TLR4^+^), or neither (TLR2^−^/TLR4^−^) were either untreated (black line) or exposed to the four different GPIs (II, III, V and VI) extracted from 1×10^9^ parasites (gray line). CD25 expression was measured by FACS analysis 18 h after stimulation. The figure is representative of three independent experiments. Percentage = [percentage of CD25 expression (M2) on GPI-stimulated cells] minus [percentage of CD25 expression (M2) on medium stimulated cells]. (**B**) Macrophages were incubated for 15, 30, or 60 min with GPI VI extracted from 4×10^8^ PTG strain parasites. Total nuclear protein content was tested using a NF-κB assay as described in Materials and Methods. The figure is a representative experiment with PTG GPI VI and similar results were obtained with GPIs II, III and V of the PTG strain. ^**^P<0.005 compared to medium control.

In previous work, we showed that all six RH strain GPIs individually added to CHO/CD14/TLR4 cells induced the expression of CD25 [36]. To determine whether the four GPIs of the PTG strain use similar TLR signaling pathways than those of the RH strain we used two NF-κB reporter cell lines, CHO/CD14/TLR2 and CHO/CD14/TLR4, co-expressing CD14 and TLR2 or TLR4, respectively. No induction of CD25 expression was observed on control cells when stimulated with individual PTG strain GPIs (Figure 5A, left panel). Low CD25 expression levels were measured when PTG strain GPIs were individually incubated with CHO/CD14/TLR2 cells (Figure 5A, middle panel). The expression of CD25 was activated significantly on CHO/CD14/TLR4 cells (Figure 5A, right panel) with magnitudes and kinetics similar to those described for the RH strain GPIs [36]. The GPI-induced TNF-α expression was previously shown to rely on the regulatory control of NF-κB p65 in macrophages [23]. Figure 5B illustrates that the GPIs of the PTG strain were also able to activate NF-κB p65 in macrophages. This result suggests that NF-κB p65 mediates the cytokine production in response to GPIs of the PTG strain. Taken together, these data indicate that RH and PTG strain GPIs have similar inflammatory capacities, mediated by comparable TLR/NF-κB-dependent signaling.

## Discussion

In the present work, we demonstrate that the GPIs of the virulent *T. gondii* RH and the avirulent PTG strains have similar structures and induce comparable biological effects in macrophages but differ in terms of total GPI-anchor content and degree of GPI-anchored protein N-glycosylation, both were significantly increased in the virulent RH strain. These findings are in line with several earlier studies that have shown that purified GPIs from protozoan parasites are able to activate immune cells of both myeloid and lymphoid origin [51]. In earlier work, we demonstrated that GPIs of RH and PTG strains bind with similar high affinities to human galectin-3 that is required to induce TNF-α secretion by macrophages in response to *T. gondii* GPIs [37]. Herein we showed that purified and individually tested GPIs from RH and PTG strains induce comparable secretion levels of TNF-α and IL-12 by macrophages, likely as a result of almost identical structures of GPI core glycans and lipid moieties. We showed further that the GPIs of the PTG strain activate TLR4 in a NF-κB CHO/CD14 reporter cell line with comparable magnitudes to those of the RH strain, suggesting that the cytokine induction is due to identical signaling events initiated by macrophage TLRs [36]. The GPIs of both strains have been also shown to stimulate the synthesis of matrix metalloproteinase (MMP)-9 by macrophages in a TNF-α-dependent manner and to degrade extracellular galectin-3 [52]. The GPI-induced degradation of galectin-3 by MMP-9 could have a regulatory function on TLR-signaling in response to GPIs [52]. We conclude that GPIs of RH and PTG strains activate similar signaling events in macrophages due to similar structures.

Structural differences between GPI core glycans of both strains may nevertheless exist since acid-labile residues, such as phosphate, phosphate glycans or phosphoethanolamine, removed by the acidic treatments are not detected. The presence of *sn-2* fatty acids (C18∶1) in the *T. gondii* GPIs found here and elsewhere [44] is reminiscent to the immunogenic GPIs in *Plasmodium falciparum* mid-schizont stages, which incorporate C18∶1 (88%) and C18∶2 (12%) at the *sn-2* position of their diacylglycerolipid [53], and in *Trypanosoma cruzi* trypomastigotes GPIs, which contain C16∶0 (37%), C18∶1 (31%) along with C18∶2 (21%) at their *sn-2* position of their alkylacylglycerolipid [54]. We reasoned that strain-specific differences in the GPI composition could be due to different amounts of protein-free GPIs and/or particular GPI-anchored surface proteins. The comparative analysis of total GPI content demonstrated that the PTG strain had significantly lower quantities of both protein-free GPIs and GPI-anchored proteins, which is likely due to extremely low levels of GPIs I and IV and not because of structural differences in the parasite form between the two strains [55]. The molecular mechanisms and the biological significance underlying the apparent lack of GPIs I and IV in the PTG strain are unknown and have yet to be established. Of further interest would be to analyze the GPI biosynthesis of *T. gondii* strains other than RH and PTG to verify whether the observed differences in the GPI content represent a general phenomenon between virulent and avirulent strain types. The bulk carbohydrate composition analysis of total GPI-anchored proteins revealed that the molar ratios of Man and GlcNAc are almost twice as high in the RH strain than in those of the PTG strain. Low molar amounts of GalNAc suggest evidence of O-glycosylation in GPI-anchored proteins. Alternatively, the fucose with xylose, galactose and possibly glucose, may form part of a yet unidentified glycan structure that may either be co-purified with the GPI-anchored proteins, or is part of a glycoprotein. In summary, these data suggest that N-linked glycosylation is more extensive in GPI-anchored proteins of the virulent RH strain than in those of the avirulent PTG strain. These results do not, however, demonstrate whether there are specific heavily N-glycosylated GPI-anchored protein(s) missing in the avirulent strain or generally there is less glycosylation of the same GPI-anchored protein(s) (Fauquenoy *et al.*, 2008). Recent work has shown that members of the SRS gene family are polymorphic, codominantly and differentially expressed as distinct, largely nonoverlapping sets of SRS antigens in a parasite strain type- and developmental-stage-specific manner [33]. The biological roles of most SRS and SUSA proteins remain largely understudied, however, some have been reported to function as virulence determinants, involved in parasite adhesion and invasion of host cells (SAG3) [56] and stimulation of host immunity (SAG1) [57], [58]. Recently it has been demonstrated that targeted deletion of the SRS29B (SAG1) and SRS34A (SAG2A) genes in the RH strain led to a specific and substantial increase in SRS29C (SRS2) protein surface expression to a level found on the surface of type 2 strain parasites, resulting in an attenuated virulence phenotype in the mouse model. It was hypothesized that decreased virulence is related to an altered immune response towards fine-tune and/or dampening the inflammatory capacity of other immunoreactive SRS proteins such as SRS29B (SAG1) [33]. Interestingly, our non-quantitative peptide-mass fingerprinting analysis of GPI-anchored proteins revealed that the most abundant parasite-specific SRS proteins, namely SAG1, SAG2, and SRS3 but not SRS2 were present in both strains, whereas the SDS-PAGE analysis of RH and PTG strain GPI-anchored protein fractions clearly showed several differences in the protein distribution pattern, which however could also reflect N-glycosylation differences. Further studies are required to identify and unravel the biological function of these differently expressed proteins in GPI-anchored protein fractions of both strains, including attempts to alter gene expression and targeted gene deletion, followed by testing their relevance in virulence and pathogenesis in the mouse model. The protein-free Glc-GalNAc-substituted GPIs were found equally present and arranged as clusters only in extracellular parasites of both RH and PTG strains. We therefore speculate that protein-free GPIs have dual functions in stimulating host immunity and/or functions in parasite adhesion of target host cells. Several studies have shown that protein-free GPIs or glycoinositolphospholipids (GIPLs) containing ceramides of protozoan parasites elicit a detrimental stimulation of the host innate immune system [51]. We have previously demonstrated that protein-free GPIs containing the Glc-GalNAc side-branch are highly immunogenic in animals and are responsible to elicit an early IgM response in humans [32], [35], [59]. Furthermore, we found that protein-free GPIs are components of DRMs with enrichments of proteins involved in parasite motility and host cell invasion [32]. Further studies are needed, however, to determine whether protein-free Glc-GalNAc-substituted GPIs have indeed dual roles in stimulating the host immunity and functions in host cell invasion as previously proposed for *T. gondii* profilin [60].

## Supporting Information

Figure S1 **Protein-free Glc-GalNAc-substituted GPIs are clustered and exclusively present on extracellular parasites.** Parasites were collected from cell culture supernatants, while intracellular parasites were mechanically released from host cells with glass beads in a Mixer Mill homogenizer. Parasites were purified, separately fixed on microscope slides with CellTack (BD Biosciences), and stained with the mAb T54 E10 as described in Materials and Methods without initial permeabilization. The mAb T4 1E5, specific for the major *T. gondii* surface protein SAG1 was used as control. Slides were examined on a Zeiss Axiophot 200M microscope equipped with ApoTome (Carl Zeiss Inc.).
(PDF)

Figure S2 **Comparison of** ***T. gondii*** **RH and PTG strain GPI-anchored protein fractions.** GPI-anchor protein fraction was obtained after the protein-free GPI extraction from the same parasite pellet by two extractions with water-ethanol-diethylether-pyridine-ammonium hydroxide (15∶15∶5∶1∶0.017, by volume). Extracts were pooled and dried under a stream of nitrogen and solubilized in Laemmli-sample buffer and boiled for 5 min. Equal amounts of GPI-anchored protein fractions of both strains were electrophoresed on 10% (w/v) SDS–PAGE and proteins were stained with Coomassie Blue. The filled arrowhead points to the expected size of SAG1 (P30) protein.
(PDF)

Table S1 **Assignment table of** **Figure 2C** **.** ES-MS analysis of the PI moieties released by deamination from purified protein-free GPIs from RH and PTG strains. ^a^All of the molecular species refer to the corresponding peaks in the negative ion mode in Figure 2C. The peaks containing the most abundant species are in bold. ^b^Observed [M-H]-ions, mass over charge from survey scans as described in the experimental procedure section. ^c^Peak identities refer to total number of carbon atoms and double bonds. ^d^Only the principal component is given. ^e^Theoretical monoisotopic masses were obtained when possible with LIPIDMAPS, otherwise they were calculated using http://www.sisweb.com/referenc/tools/exactmass.htm.
(PDF)

Table S2 **Analysis of RH and PTG strain GPI-anchored proteins.** Results obtained from mass-fingerprinting of a fraction containing purified GPI-anchored proteins from both RH and PTG strains. The GPI-anchor protein fraction was obtained after the protein-free GPI extraction from the same parasite pellet by two extractions with water-ethanol-diethylether-pyridine-ammonium hydroxide (15∶15∶5∶1∶0.017, by volume). The supernatant was dried under a stream of nitrogen and proteins were re-suspended in trypsin digestion buffer [12.5 mg/ml modified bovine trypsin (Roche), 0.1% *n*-octyl glucoside (Calbiochem), 20 mM ammonium bicarbonate]. An equal volume of acetonitrile/0.3% trifluoroacetic acid was added to extract the peptides. MALDI mass spectra were generated using a Voyager DE-STR MALDI-TOF MS system (PerSeptive Biosystems) with delayed extraction in the reflectron mode. Protein identification was from a comparison of peak list data generated from the Data Explorer application (PerSeptive Biosystems) against NCBInr (non-redundant) and Swissprot databases using the Protein-Prospector V3.4.1 software MS-Fit (http://www.prospector.ucsf.edu). Only hits with a high score are given so that a very high confidence level could be maintained, with major surface proteins in bold.
(PDF)
