# Supplementary figures and images for: Virulent and Avirulent Strains of Toxoplasma gondii Which Differ in Their Glycosylphosphatidylinositol Content Induce Similar Biological Functions in Macrophages
Source: PLoS One. 2014 Jan 28;9(1):e85386. doi: 10.1371/journal.pone.0085386 (PMC3904843; doi:10.1371/journal.pone.0085386)

Figure S1

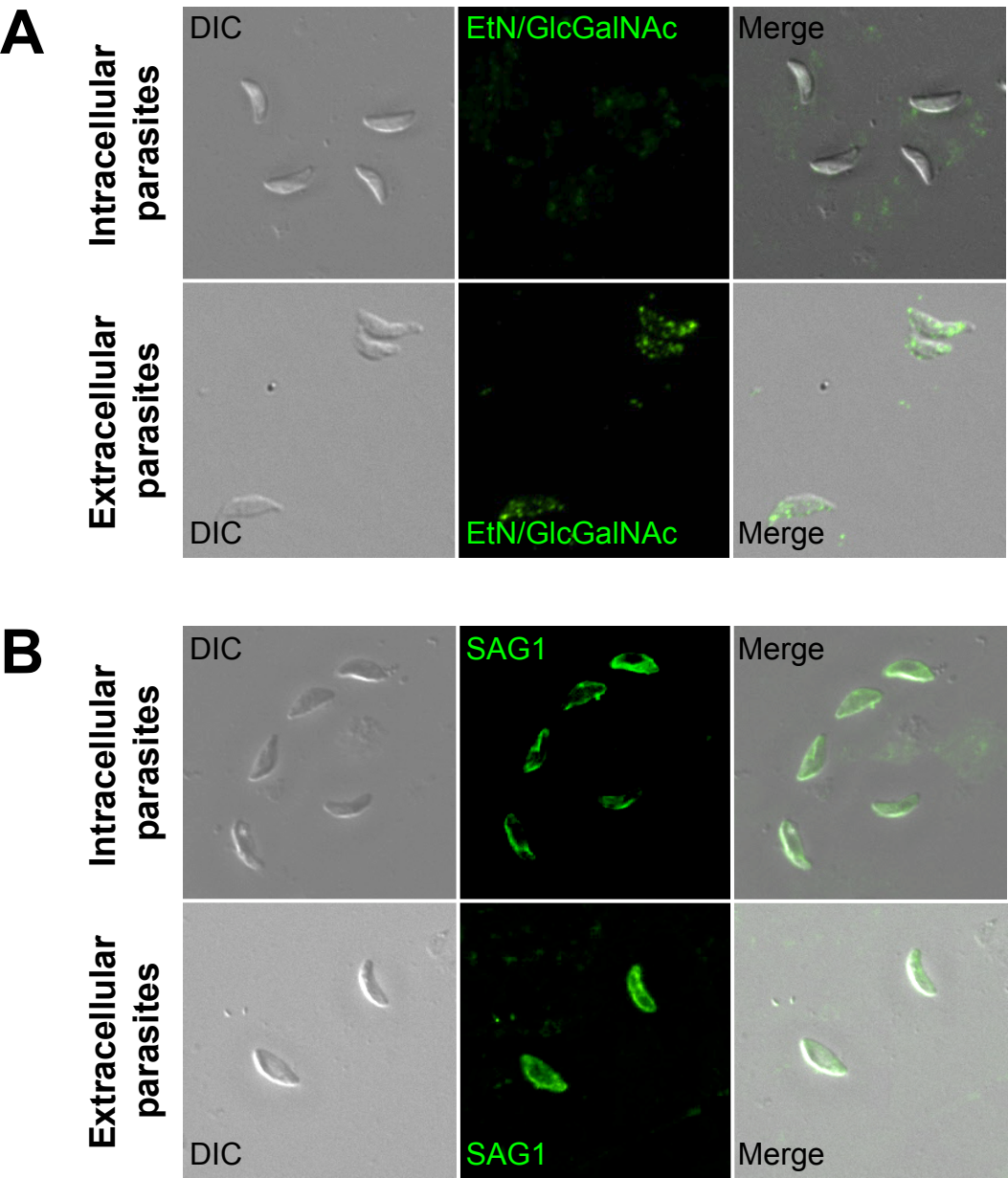

Supplement: Figure S1 — Protein-free Glc-GalNAc-substituted GPIs are clustered and exclusively present on extracellular parasites. Parasites were collected from cell culture supernatants, while intracellular parasites were mechanically released from host cells with glass beads in a Mixer Mill homogenizer. Parasites were purified, separately fixed on microscope slides with CellTack (BD Biosciences), and stained with the mAb T54 E10 as described in Materials and Methods without initial permeabilization. The mAb T4 1E5, specific for the major T. gondii surface protein SAG1 was used as control. Slides were examined on a Zeiss Axiophot 200M microscope equipped with ApoTome (Carl Zeiss Inc.). (PDF) [file pone.0085386.s001.pdf]

Figure S2

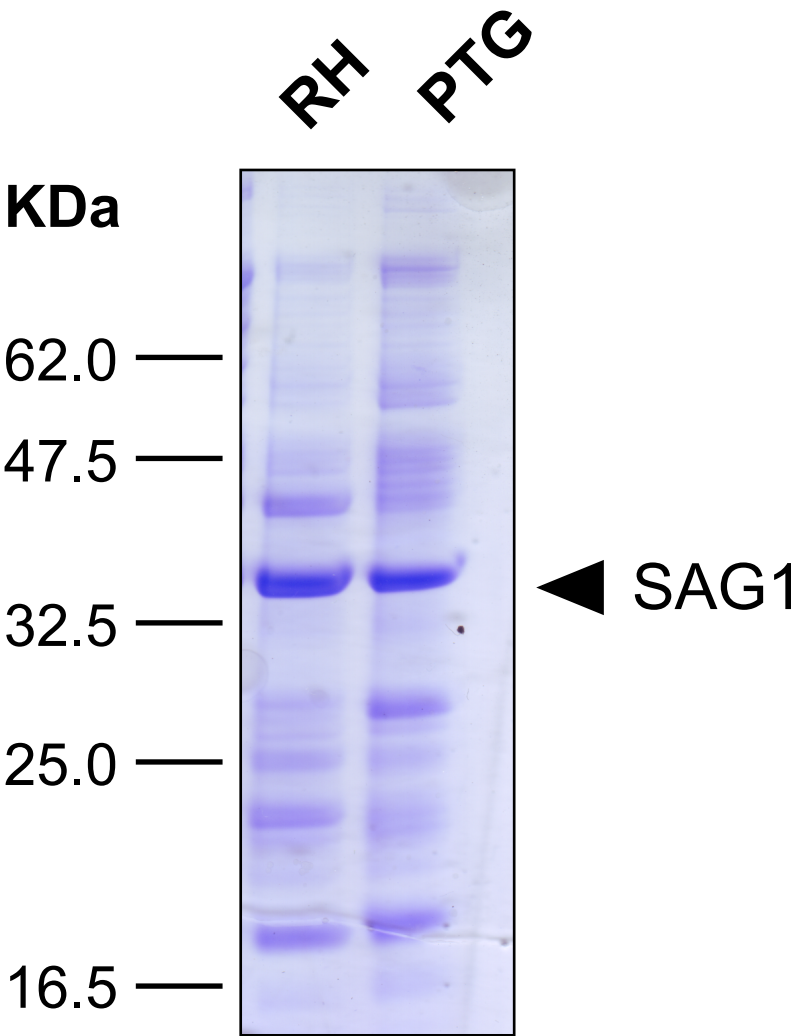

Supplement: Figure S2 — Comparison of T. gondii RH and PTG strain GPI-anchored protein fractions. GPI-anchor protein fraction was obtained after the protein-free GPI extraction from the same parasite pellet by two extractions with water-ethanol-diethylether-pyridine-ammonium hydroxide (15∶15∶5∶1∶0.017, by volume). Extracts were pooled and dried under a stream of nitrogen and solubilized in Laemmli-sample buffer and boiled for 5 min. Equal amounts of GPI-anchored protein fractions of both strains were electrophoresed on 10% (w/v) SDS–PAGE and proteins were stained with Coomassie Blue. The filled arrowhead points to the expected size of SAG1 (P30) protein. (PDF) [file pone.0085386.s002.pdf]
