# Supplementary material for: Virulent and Avirulent Strains of Toxoplasma gondii Which Differ in Their Glycosylphosphatidylinositol Content Induce Similar Biological Functions in Macrophages
Source: PLoS One. 2014 Jan 28;9(1):e85386. doi: 10.1371/journal.pone.0085386 (PMC3904843; doi:10.1371/journal.pone.0085386)
Supplement: Table S1 — Assignment table of Figure 2C . ES-MS analysis of the PI moieties released by deamination from purified protein-free GPIs from RH and PTG strains. aAll of the molecular species refer to the corresponding peaks in the negative ion mode in Figure 2C. The peaks containing the most abundant species are in bold. bObserved [M-H]-ions, mass over charge from survey scans as described in the experimental procedure section. cPeak identities refer to total number of carbon atoms and double bonds. dOnly the principal component is given. eTheoretical monoisotopic masses were obtained when possible with LIPIDMAPS, otherwise they were calculated using http://www.sisweb.com/referenc/tools/exactmass.htm. (PDF) [file pone.0085386.s003.pdf]

Table S1

**Observed** ***m/z***[ab]	**Lipid**[c]	**Principal component**[d]	**Theoretical** ***m/z***[e]
752.5	28:1	14:0/14:1	752.45
754.6	28:0	14:0/14:0	754.46
778.6	30:2	14:1/16:1	778.46
780.3	30:1	14:0/16:1	780.48
782.2	30:0	14:0/16:0	782.49
804.5	32:3	14:1/18:2	804.48
806.9	32:2	14:1/18:1	806.49
808.3	32:1	14:0/18:1	808.51
**833.4**	**34:2**	**16:1/18:1**	**833.52**
**835.5**	**34:1**	**16:0/18:1**	**835.53**
837.5	34:0	16:0/18:0	837.55
859.5	36:3	18:2/18:1	859.53
**861.5**	**36:2**	**18:1/18:1**	**861.55**
**863.5**	**36:1**	**18:0/18:1**	**863.57**
883.4	38:5	18:1/18:1	883.53
885.5	38:4	18:0/18:1	885.55
**887.3**	**38:3**	**18:1/18:1**	**887.57**
**889.7**	**38:2**	**20:1/18:1**	**889.58**
917.6	40:2	22:0/18:2	917.61
919.6	40:1	22:0/18:1	919.63
