# Supplementary material for: Virulent and Avirulent Strains of Toxoplasma gondii Which Differ in Their Glycosylphosphatidylinositol Content Induce Similar Biological Functions in Macrophages
Source: PLoS One. 2014 Jan 28;9(1):e85386. doi: 10.1371/journal.pone.0085386 (PMC3904843; doi:10.1371/journal.pone.0085386)
Supplement: Table S2 — Analysis of RH and PTG strain GPI-anchored proteins. Results obtained from mass-fingerprinting of a fraction containing purified GPI-anchored proteins from both RH and PTG strains. The GPI-anchor protein fraction was obtained after the protein-free GPI extraction from the same parasite pellet by two extractions with water-ethanol-diethylether-pyridine-ammonium hydroxide (15∶15∶5∶1∶0.017, by volume). The supernatant was dried under a stream of nitrogen and proteins were re-suspended in trypsin digestion buffer [12.5 mg/ml modified bovine trypsin (Roche), 0.1% n-octyl glucoside (Calbiochem), 20 mM ammonium bicarbonate]. An equal volume of acetonitrile/0.3% trifluoroacetic acid was added to extract the peptides. MALDI mass spectra were generated using a Voyager DE-STR MALDI-TOF MS system (PerSeptive Biosystems) with delayed extraction in the reflectron mode. Protein identification was from a comparison of peak list data generated from the Data Explorer application (PerSeptive Biosystems) against NCBInr (non-redundant) and Swissprot databases using the Protein-Prospector V3.4.1 software MS-Fit (http://www.prospector.ucsf.edu). Only hits with a high score are given so that a very high confidence level could be maintained, with major surface proteins in bold. (PDF) [file pone.0085386.s004.pdf]

Table S2

Strain	Accession	Definition	Mass	Score	Queries
RH	O60955	14-3-3 protein homologue	30679	412	40
	P13404	Dense granule protein 2 (GRA2) precursor	19830	96	17
	O00933	Dense granule protein 7 (GRA7) precursor	25842	152	10
	Q9GRG4	MIC3 microneme protein precursor	37859	96	4
	Q9GSV5	MIC10 microneme protein precursor	23109	151	7
	Q8IT73	MIC11 microneme protein precursor	22403	149	12
	Q95UJ7	MLC1 myosin light chain	24108	103	4
	**O15694**	**SAG1-related sequence 3**	**36215**	**94**	**1**
	**Q7KNK3**	**Surface antigen P30**	**26617**	**162**	**7**
	**Q9NBH0**	**Surface antigen P22**	**19094**	**160**	**5**
	Q9NJS3	Tachyzoite serine proteinase inhibitor	32569	111	5
PTG	O60955	14-3-3 protein homologue	30679	218	34
	O77470	Acyl carrier protein precursor	19368	93	2
	**O15681**	**Apical membrane antigen 1 homolog**	**59940**	**82**	**5**
	P13403	Dense granule protein 1 (GRA1, p24) precursor	20137	63	8
	P13404	Dense granule protein 2 (GRA2) precursor	19830	177	27
	P13404	**Dense granule protein 2 (GRA2)**	**28018**	**164**	**26**
	Q07828	Dense granule protein 5 (GRA5)	12969	99	9
	Q27003	Dense granule protein 6 (GRA6, p32, p33)	24015	87	3
	O00933	Dense granule protein 7 (GRA7) precursor	25347	232	11
	Q9BPL7	Enolase 2 (2-phosphoglycerate dehydratase 2)	48259	73	4
	Q7Z289	Gliding-associated protein 45	27301	71	5
	Q9BKE2	Glyceraldehyde-3-phosphate dehydrogenase	36605	97	5
	P90611	H4 protein	25968	95	2
	O00834	MIC1 microneme protein	48642	80	1
	Q9GRG4	MIC3 microneme protein precursor	37859	111	5
	Q9GSV5	MIC10 microneme protein precursor	23109	187	18
	Q8IT73	MIC11 microneme protein precursor	22403	109	15
	Q95UJ7	Myosin light chain TgMLC1	24108	132	7
	Q9XZD5	Peroxisomal catalase	57234	94	11
	Q9XZD5	Peroxisomal catalase	57234	92	10
	Q8MPF4	Putative PDI-like protein	24894	64	3
	**O15694**	**SAG1-related sequence 3**	**36215**	**100**	**2**
	Q95UQ2	Subtilisin-like protein	85034	117	15
	**Q9NBH1**	**Surface antigen P22**	**19010**	**133**	**4**
	**Q6DNI7**	**Surface antigen P30**	**34806**	**183**	**5**
	Q9NG25	Toxofilin	27115	133	7
